# Quantitative Fluorescence Imaging of Chemophototherapy Drug Pharmacokinetics Using Laparoscopic SFDI

**DOI:** 10.3390/ijms26125571

**Published:** 2025-06-11

**Authors:** Rasel Ahmmed, Elias Kluiszo, Semra Aygun-Sunar, Matthew Willadsen, Hilliard L. Kutscher, Jonathan F. Lovell, Ulas Sunar

**Affiliations:** 1 Department of Biomedical, Engineering, Stony Brook University, Stony Brook, NY 11794, USA; rasel.ahmmed@stonybrook.edu (R.A.); elias.kluiszo@stonybrook.edu (E.K.); semra.aygun@stonybrook.edu (S.A.-S.); 2 POP Biotechnologies, Buffalo, NY 14228, USA; mwilladsen@popbiotech.com (M.W.); hkutscher@popbiotech.com (H.L.K.); 3 Department of Biomedical Engineering, University at Buffalo, Buffalo, NY 14260, USA; jflovell@buffalo.edu

**Keywords:** laparoscope, spatial frequency domain imaging, quantitative fluorescence imaging, doxorubicin drug concentration, light-triggered release, porphyrins, photodynamic therapy

## Abstract

Chemophototherapy (CPT) is an emerging cancer treatment that leverages the synergistic effects of photodynamic therapy (PDT) and chemotherapy. This approach utilizes photosensitizers like Porphyrin-Phospholipid (PoP) and combined with chemotherapeutic like Doxorubicin (Dox) to enable light-triggered drug release and targeted tumor destruction. Here, we present the validation of a wide-field laparoscopic spatial frequency domain imaging (SFDI) system in an ovarian cancer model. The system allows quantitative fluorescence imaging to obtain absolute drug concentrations in vivo to obtain the absolute concentrations of PoP and Dox fluorescence by correcting for tissue absorption and scattering effects. Fluorescence imaging revealed a significant reduction (~25%, *p* < 0.001) in PoP concentration in tumor regions post-illumination, demonstrating PDT-mediated photobleaching. Next, the Dox release experiment showed an increase of ~13 µg/mL Dox concentration at the local site. The ability to quantify both PoP and Dox fluorescence concentrations with a laparoscopic system underscores its potential for intraoperative monitoring of CPT efficacy. These findings indicate wide-field laparoscopic SFDI as a promising tool for guiding minimally invasive PDT and targeted drug delivery in preclinical and future clinical settings.

## 1. Introduction

Ovarian cancer remains one of the most lethal gynecological malignancies due to its high recurrence rates and peritoneal micrometastases, which are challenging to detect and treat effectively. Even after surgical treatment and adjuvant chemotherapy, up to 60% of patients may still exhibit occult disseminated ovarian disease [1,2,3], and systemic chemotherapy often leads to significant toxic side effects [4,5,6]. In addition, traditional imaging techniques, such as computed tomography (CT), magnetic resonance imaging (MRI), positron emission tomography (PET), and ultrasound, demonstrate less sensitive detection than reassessment surgeries [3,7]. Thus, an unmet need exists for both sensitive imaging technologies for improved detection and therapeutic approaches that selectively treat superficial tumors in the intraperitoneal cavity while avoiding healthy tissue.

Photodynamic therapy (PDT) has gained significant attention as a minimally invasive cancer treatment modality that leverages light, a photosensitizing agent, and molecular oxygen to generate cytotoxic reactive oxygen species (ROS) capable of selectively destroying tumor cells [8,9]. Porphyrin-based compounds (photosensitizers—PS) are used for PDT and imaging because they strongly fluoresce, preferentially accumulate ~2–3 fold in malignant cells, and have demonstrated some success in detecting subcutaneous tumors with qualitative imaging approaches [6,10,11,12,13,14]. Porphyrin conjugates (Porphyrin-phospholipid—PoP) have been through over 10 human phototherapy clinical trials. Because superficial tumors can be on the surface and accessible via surgery, PDT is an attractive approach for fulfilling the adjunctive therapy role of surgery [15]. However, high normal tissue toxicity has been a major obstacle to the clinical translation of PDT for ovarian cancer. Seminal works by Hasan [5,6,16,17,18,19] and Mordon [1,20,21] groups have shown epidermal growth factor receptor (EGFR) and folate-targeted approaches reducing the peritoneal tissue toxicity of PDT by using semi-quantitative imaging approaches in animal models. Liposomal nanocarriers serve to improve the biodistribution and efficacy of a variety of cancer drugs, with the lipid envelope protecting drugs from degradation inside the body and allowing for passive diffusion into tumor sites via the enhanced permeability and retention (EPR) effect [13,22]. While liposomal formulations can decrease side effects, drug delivery to discrete tumor sites is suboptimal from intravenous administration alone [23]. The light-activated PoP-liposomes used here [24,25] improve the concentration of drug delivered to the tumor site by selectively permeabilizing the PoP liposomes in the presence of NIR (~660 nm) [26,27,28]. Both PoP and Doxorubicin (Dox) are inherently fluorescent, so fluorescent imaging of the tumor site before and after light activation can quantify drug kinetics in vivo [29]. We use PoP fluorescence to localize the tumor in vivo due to its longer fluorescence wavelength (~720 nm vs. 590 nm), and then Dox fluorescence allows assessing local Dox release at the targets.

To develop an image-guided system for the delivery and detection of PS, knowledge about PS concentrations at and near the target cells is essential. Our approach capitalizes on the high sensitivity and specificity of fluorescence contrast, the fact that PoP is highly fluorescent [30], and the increased resolution of wide-field imaging. However, the accurate quantification of drug concentrations in tumors remains an ongoing challenge because the raw fluorescence signal is affected by tumor absorption and scattering properties, which confound the true fluorescence contrast [31,32]. To correct this distortion, spectroscopic measurements can be taken to quantify tissue attenuation, but it is point-specific [33,34,35,36]. SFDI gathers tissue data over a wide area, allowing for accurate measurements in heterogeneous tissue like the intraperitoneal cavity.

SFDI can quantify both optical absorption and scattering during reflectance imaging mode [6] and is easy to implement [37]. Knowledge of the optical parameters can allow the modeling of the light dose distribution within the treatment field. In addition to light dosimetry, these parameters can provide the fluorescence correction factor for tissue attenuation so that one can extract absolute fluorescence concentration in vivo.

We employed a custom laparoscopic SFDI system to quantify the absolute concentration of PoP and Dox fluorescence concentrations in an ovarian cancer model. The fluorescence of Doxorubicin (Dox), which is embedded in the liposomal construct of PoP liposomes enables spatially and temporally controlled release of Dox from liposomes using near-infrared light [38]. The quantification of PoP fluorescence concentration revealed a significant photobleaching effect compared to the periphery.

## 2. Results

### 2.1. PoP Fluorescence Concentration Contrast and Photobleaching in Subcutaneous Tumors

**Figure 1:**
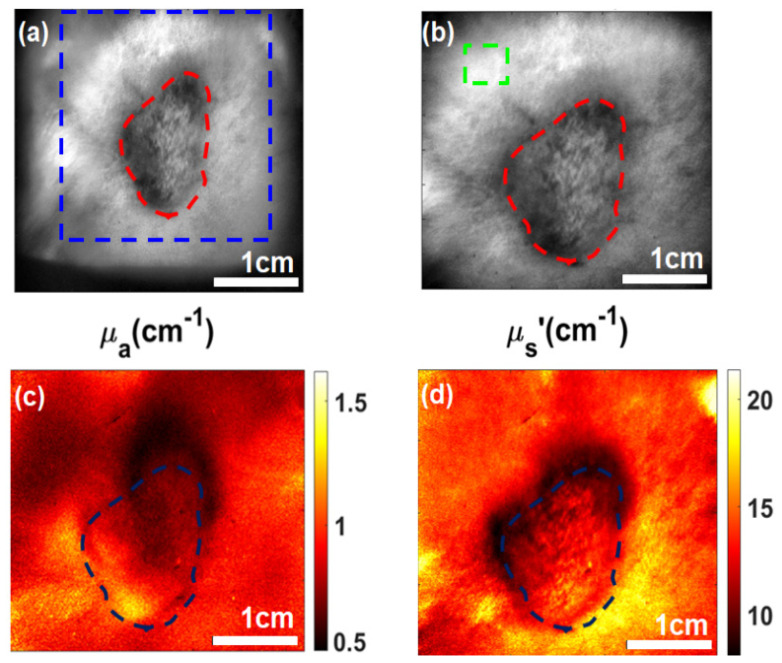
Representation of the optical properties of the tumor and periphery: (**a**) Raw image with a specific region of interest (ROI) including both the periphery and the tumor region. (**b**) Diffuse reflectance image with the lesion and periphery marked. (**c**,**d**) Absorption and scattering map for 660 nm.

Figure 1 provides a comprehensive visualization of the optical characteristics of the tumor and its surrounding periphery. Figure 1a,b show the diffuse reflectance image with the lesion and the surrounding periphery area. These images offer insight into the spatial variations in light scattering and absorption within the tissue, whereas Figure 1c and Figure 1d show the absorption and scattering maps at 660 nm, respectively. Figure 1c showed higher absorption at the lesion compared to the surrounding periphery likely due to enhanced vascularization or chromophore concentration in the tumor. Conversely, the scattering parameter at 660 nm was lower at the tumor in Figure 1d suggesting structural alterations such as reduced collagen density or cellular disorganization. The 660 nm wavelength was chosen because this is the excitation wavelength of PoP used for PDT that also results in Dox release.

**Figure 2:**
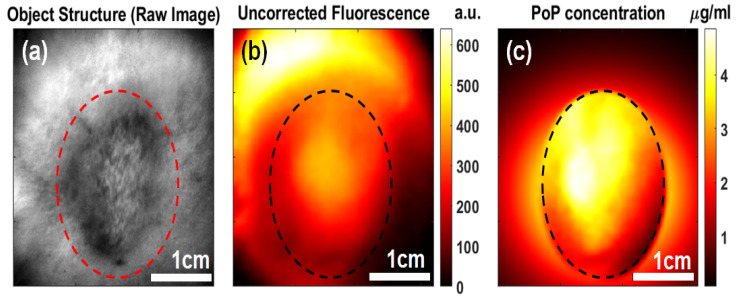
Representative images of a tumor after light administration to PoP: (**a**) White light structural image showing the tumor area. (**b**) An uncorrected fluorescence image does not show localized contrast. (**c**) PoP fluorescence concentration indicated a higher contrast between the tumor and the surrounding area compared to the uncorrected fluorescence.

Figure 2 shows the representative images of a tumor in a mouse 1 h after the administration of PoP. Figure 2a shows the white light image to indicate the tumor location. The tumors were large (~9 mm diameter). The uncorrected fluorescence shows unexpected tumor contrast (705.1 ± 94.2 a.u.-tumor vs. 983.1 ± 58.5 a.u.-periphery). This suggests that raw fluorescence intensity alone does not accurately reflect PoP distribution due to light attenuation in tissue. However, after applying a correction algorithm to quantify fluorescence, the quantified absolute PoP concentration image demonstrated a higher contrast in the tumor compared to peripheral tissue (0.24 ± 0.03 µg/mL vs. 0.18 ± 0.01 µg/mL, respectively). This corrected fluorescence concentration confirms that PoP preferentially accumulates in the tumor, enhancing tumor contrast and supporting its potential role in targeted photodynamic therapy (PDT) and drug delivery applications.

**Figure 3:**
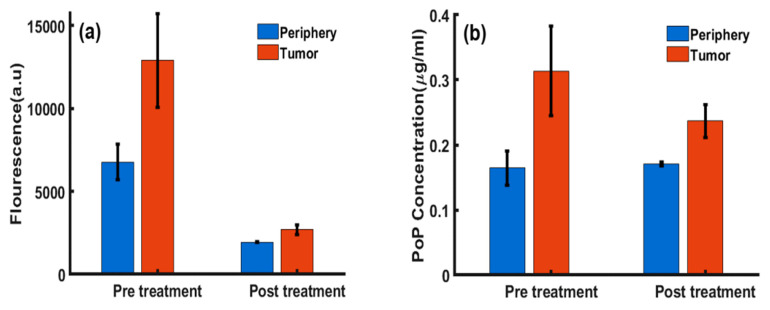
Extracted parameters from tumor and periphery pre- and post-PDT: (**a**) Bar plot showing the comparison of PoP fluorescence in the tumor vs. peripheral tissue pre- and post-treatment light. (**b**) Comparison of the PoP concentration (µg/mL) pre- and post-treatment.

We also quantified the PS photobleaching in vivo. As shown in Figure 3a, the PoP fluorescence amplitude was approximately 2.2 times higher in tumor tissue than in normal tissue before PDT. Following treatment with light irradiation, fluorescence imaging revealed a significant (~75%, with *p* < 0.001) reduction in PoP concentration in the tumor region due to photobleaching [39,40,41,42]. Figure 3b shows the absolute PoP concentration, indicating greater PoP uptake in tumor tissue compared to normal tissue, with a tumor-to-normal tissue uptake ratio of approximately 1.7, consistent with previously reported values [7]. After PDT, the PoP concentration in the tumor decreased by about ~25% (with *p* < 0.001) due to photobleaching after treatment, while the drug concentration in normal tissue remained like the pre-PDT value.

### 2.2. Dox Release in Mouse Carcass

#### 2.2.1. Dox Imaging Contrast

**Figure 4:**
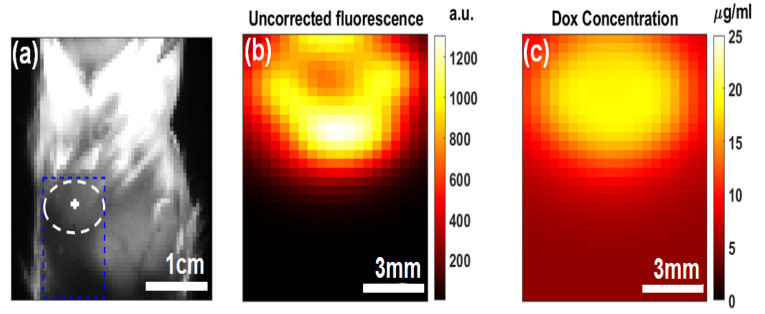
Representative Dox quantification in a mouse carcass: (**a**) White light image showing the structure of the PoP injection and release site. (**b**) Uncorrected Dox fluorescence image after 8 min illumination. (**c**) Dox concentration image after 8 min illumination.

We then investigated Dox imaging contrast in a BALB/c mouse carcass. Figure 4a highlights the white light structural image, marking the PoP injection and Dox release sites. The injection site was exposed to treatment light for 8 min. The uncorrected fluorescence in Figure 4b and the concentration map in Figure 4c revealed differences in image contrast. This is due to attenuation correction in the tissue’s optical properties. Additionally, autofluorescence was subtracted from the total fluorescence signal to isolate the Dox contribution. Autofluorescence accounted for approximately 60% of the initial background signal before treatment and about 10% of the peak fluorescence signal during treatment.

#### 2.2.2. Dox Release Kinetics

**Figure 5:**
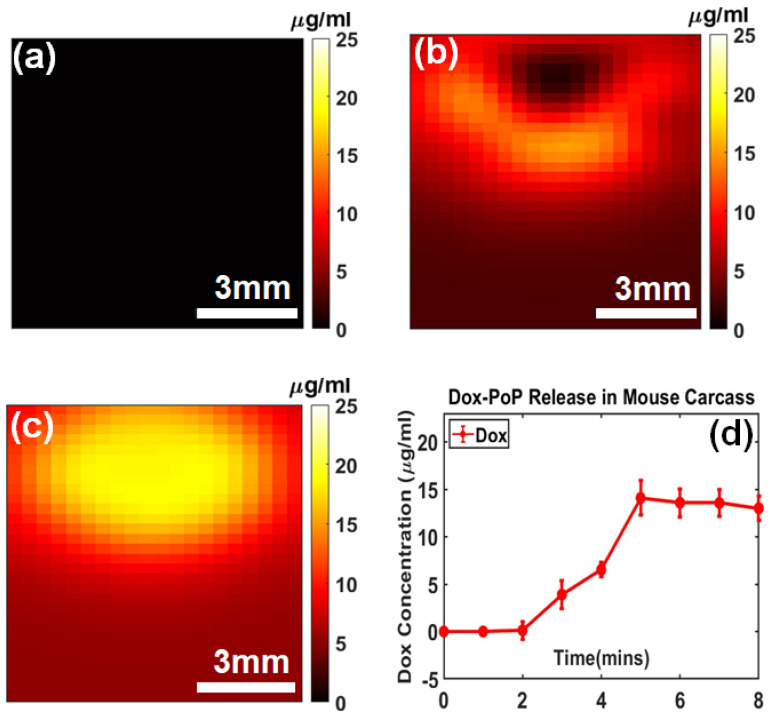
Dox release kinetics in a mouse carcass: (**a**) Pre-treatment, (**b**) 4 min post-treatment, and (**c**) 8 min post-treatment. (**d**) Complete Dox release kinetics curve with the mean and standard deviation of the ROI.

We examined Dox release kinetics in a mouse carcass as shown in Figure 5. Figure 5a, Figure 5b and Figure 5c display the Dox concentrations at pre-treatment, 4 min post-treatment, and 8 min post-treatment, respectively, showing an increase from 6.53 ± 0.74 µg/mL to 13.01 ± 1.24 µg/mL distribution, likely due to variations in optical parameters, particularly scattering differences between the skin and intralipid^®^. This is likely attributed to the presence of overlying skin and the limited penetration depth of the excitation light. Additionally, it is expected that the treatment light did not fully probe the entire drug volume due to the partial volume effect caused by the skin layer and the uneven distribution of the Dox-PoP liposome beneath the skin. As a result, a portion of the total volume near the surface may have been released initially and subsequently diffused into the untreated regions over time.

#### 2.2.3. Porphyrin Photobleaching in Dox-PoP Liposomes

**Figure 6:**
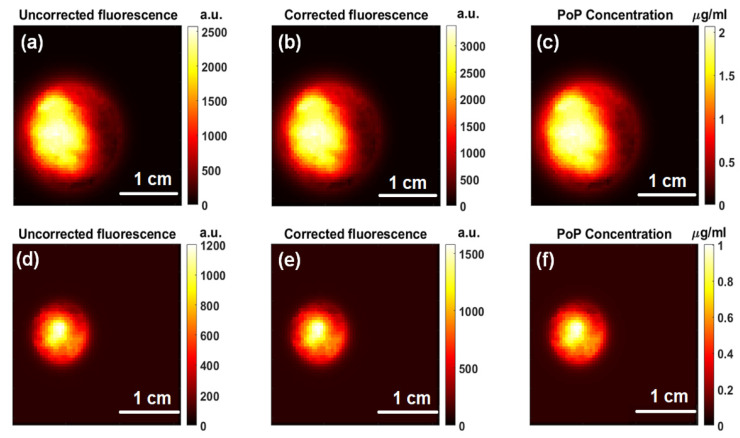
Porphyrin fluorescence at pre- and post-light treatment: (**a**–**c**) pre-treatment fluorescence and subsequent PoP concentration and (**d**–**f**) post-treatment fluorescence and subsequent PoP concentration.

We compared the PoP fluorescence before (Figure 6a–c) and after (Figure 6d–f) treatment light for Doxorubicin release. It showed a decrease from 1.57 ± 0.37 µg/mL to 0.73 ± 0.14 µg/mL, likely due to photobleaching from the strong treatment light at the excitation wavelength of the PoP. These findings confirm that the treatment light not only facilitates Dox release but also impacts PoP fluorescence (photobleaching), highlighting the importance of optimizing light dosimetry for controlled drug activation and optimizing photobleaching for the synergistic effect of chemotherapy and photodynamic therapy (PDT).

## 3. Discussion

In this study, our quantitative, wide-field laparoscopic approach uses both reflectance and fluorescence imaging modes to monitor PoP photosensitizer and Dox chemodrug. SFDI quantified the optical parameters of the model tissue to provide *a priori* information for correcting fluorescence signal attenuation to obtain absolute drug concentrations. Quantitatively derived distributions of both PoP and Dox guide CPT treatment. PoP fluorescence first indicates targeted tumor localization, while its photobleaching serves as a marker of PDT effectiveness. Dox fluorescence reveals the bioavailability of the chemotherapeutic agent within tissue.

However, achieving concurrent measurements during PDT remains challenging. Unlike probe-based methods [25], which may interrupt treatment light and require fine adjustments during measurements, wide-field SFDI avoids physical interference with the treatment area. Furthermore, non-contact methods such as SFDI dual-channel endoscope systems [24] provide the flexibility to monitor tumor treatments with a small field of view (~1 cm^2^) of superficial surfaces.

PoP fluorescence correction was significant at the tumor site, where absorption and scattering parameters were ~40% and ~15% higher than the periphery, respectively (Figure 2b,c). Without correction, raw fluorescence images could misleadingly suggest lower PoP concentration at the tumor site than at the periphery, potentially resulting in an underestimation of drug delivery. Therefore, fluorescence correction is critical for the accurate and individualized assessment of treatment efficacy.

The 660 ± 10 nm treatment light activation was generated by a laser source and collimated through an SMA fiber to activate PoP at the treatment site. In areas with highly heterogeneous optical properties, this can lead to significant variations in local light dose [43]. Thus, the knowledge of the tissue optical properties is important for both accurate drug dose distribution at pre-, during, and post-treatment, as well as accurate local treatment light delivery and optimization on an individual, tumor-by-tumor basis. The digital micromirror device (DMD) in the system can also be used during the treatment mode to modulate the spatial intensity and shape of the light, allowing customized and optimal illumination for each tumor while maintaining the necessary fluence rate for Dox-PoP liposome activation.

In the laparoscopic SFDI data, it is revealed that Doxorubicin fluorescence occurred within 6 min of treatment time, likely due to the complete release of the PoP-liposomes. Notably, the total activated Dox concentration shown in Figure 5d did not reach the injected amount (equivalent to ~23 µg/mL of Dox). This is likely due to the shallow penetration depth and partial volume effect issues in vivo. The limited penetration depth of 490 nm light might also explain the heterogeneity of the Doxorubicin signal in Figure 5b, as liposomes closer to the surface of the injection could be released first and then gradually mixed with the untreated volume over a longer period.

In Figure 5, the targeted region was located at or near the surface, allowing for direct and efficient light delivery to achieve localized drug activation. However, in deeper anatomical sites, such as the peritoneal cavity, achieving uniform light exposure over a large area can be challenging. Alternative light delivery methods [44], including interstitial fiber-optic probes [25] or endoscopic-based illumination [24], offer promising solutions for delivering targeted and efficient light for photo-triggered drug release in hard-to-reach regions.

Overall, our SFDI system has shown ~10% maximum errors in quantifying the optical properties of tissue-mimicking phantoms. This error ultimately translates to the concentration maps of fluorophores, which require the absorption and scattering parameters at the excitation and emission wavelengths as inputs. To optimize SFDI measurements in vivo, future studies will incorporate profilometry techniques to account for the curvature of the tumor site. We did not correct for the surface angle variations since the injection and tumor size were relatively small, but the error is likely still present. For larger tumors, we would need to implement a model-based Lambertian reflectance approach for profile correction, as demonstrated by Gioux et al. [45].

It should also be noted that all the reported values were obtained via post-processing with custom MATLAB (R2023b) software that fit the data to the model at each pixel. This process took between 5 and 10 min depending on the pixel binning, and was performed outside the experimental site. SFDI data acquisition took ~1 min, with 1–2 min required per wavelength for property extraction, and 2–3 min for multi-wavelength fitting. These durations could be reduced by decreasing spatial resolution (i.e., pixel binning). Utilizing a faster acquisition technique, such as a single snapshot [45], or a faster processing technique, such as the lookup table (LUT) [46] model recently proposed by Angelo et al., could reduce the overall quantification time and provide results in the clinic. This near-real-time feedback would be particularly useful for monitoring light-based therapies such as laser or photodynamic therapy.

## 4. Materials and Methods

### 4.1. Wide-Field SFDI System

**Figure 7:**
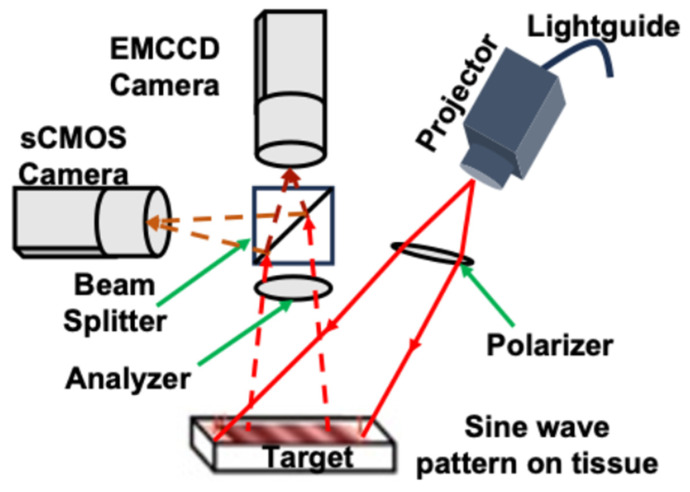
Schematic diagram of the wide-field imaging head showing the projector module; one charge-coupled device (CCD); and one sCMOS camera, beam splitter, polarizer, and analyzer. Light-emitting diode (LED) light is delivered with a light guide. Four LEDs are switched sequentially. Digital micromirror device generates sinusoidal patterns, which are projected onto the skin surface by the projector, and the reflected signal is detected by CCD cameras.

A clinic-friendly SFDI system was constructed as shown in Figure 7. The system utilized four high-power, compact light-emitting diodes (LEDs) from the LCS series, each emitting light at 590 nm, 625 nm, 660 nm, and 740 nm (Mightex, Toronto, ON, Canada). A four-channel LED controller (Mightex) sequentially activated the selected excitation wavelength. The light was directed via a liquid light guide to a projector (Light Commander; Logic PD, Inc., Minneapolis, MN, USA) equipped with a digital micromirror device (DMD) module offering a resolution of 1024 × 768 pixels.

The DMD module generated sinusoidal patterns with three distinct phases (0, 2π/3, and 4π/3) and 22 spatial frequencies ranging from 0 to 3.1764 cm^−1^. These patterns were projected onto the tumor surface, and the reflected light was captured by the cameras. The cameras were aligned to focus on the same field of view as the projector, covering an area of 22 × 22 mm^2^. A rigid light shield with an imaging window was used to block ambient light and maintain a consistent distance from the target tissue. For imaging, the system incorporated two cameras positioned on either side of a 685 nm dichroic mirror (67-085; Edmund Optics, Barrington, NJ, USA). This design ensured the precise imaging of the illuminated area. Fluorescence and reflectance imaging were performed simultaneously. The first sCMOS camera (Zyla Andor, Belfast, Ireland) captured reflectance images at 590, 630, and 660 nm, while a highly sensitive Electron-Multiplying Charge-Coupled Device (EMCCD) camera (Luca; Andor, Belfast, Ireland) acquired reflectance images at 660 nm and 740 nm and fluorescence data at 740 nm. Splitting the light at the 685 nm dichroic mirror allows for the analysis of the projected excitation light by the sCMOS and the fluorescent signal by the more sensitive EMCCD camera.

The cameras operated with an acquisition time of 100 ms per image, resulting in a total acquisition time of 27 s (calculated as 100 ms × 3 phases × 22 frequencies × 4 wavelengths). The system was fully automated using a custom LabView (version 2023 Q3, National Instruments, Austin, TX, USA) software, which included subprograms to manage all the system components. The software enabled the automatic adjustments of LED intensities and exposure times for individual subjects. To minimize specular reflections during reflectance imaging, cross-polarizers were placed in front of the projector and cameras. The LED light source intensity was maintained at <1 mW/cm^2^ to ensure safety.

The optical absorption and scattering properties were quantified by fitting a modified frequency-dependent Monte Carlo model to the measured reflectance data across multiple spatial frequencies. This process utilized a reference phantom with known optical properties, as previously described [47]. All 22 spatial frequencies, ranging from 0 to 3.18 cm^−1^, were included in the analysis. For each spatial frequency and wavelength, the three phase-shifted reflectance images were demodulated to isolate the spatially modulated component of the diffuse reflectance. The demodulated reflectance, which varies as a function of spatial modulation frequency, exhibits differing sensitivities to absorption and scattering parameters depending on the frequency. This allows SFDI to independently and accurately quantify both absorption and scattering. Using this approach, pixel-by-pixel fitting was performed to generate spatial maps of absorption and scattering properties.

### 4.2. Laparoscopic SFDI System

**Figure 8:**
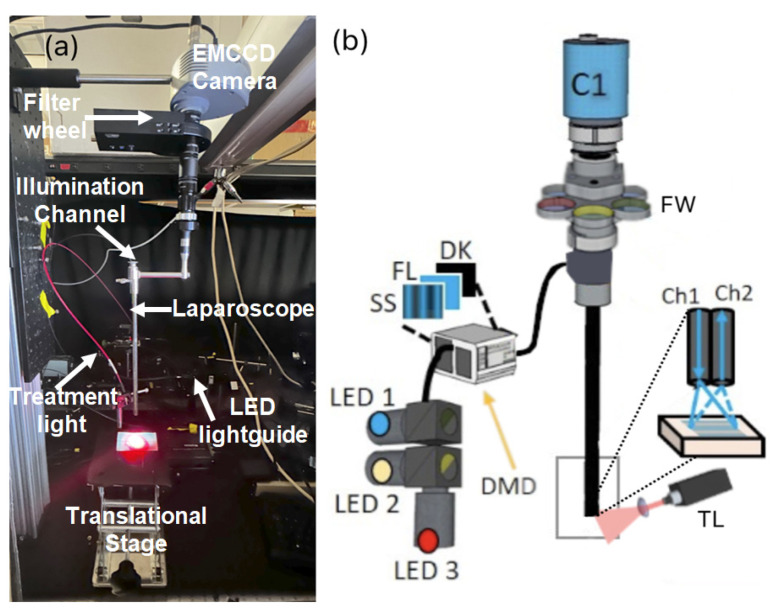
The experimental setup of laparoscopy SFDI for Doxorubicin release in mouse carcass: (**a**) laparoscope projecting the 490 nm excitation light in blue and the 656 nm treatment light in red and (**b**) laparoscope setup, showing LEDs and DMD with sinusoidal, fluorescent, and dark images projected, as well as the camera (C1), filter wheel placement (FW), treatment light (TL), and the projection (Ch1) and imaging (Ch2) channels.

The laparoscopic SFDI system was constructed as shown in Figure 8. Figure 8a shows the mouse carcass imaging setup, and Figure 8b details the projection and imaging components within the laparoscopic system.

Our laparoscopic fluorescence imaging and SFDI setup consisted of a modified DMD (LightCrafter 4500, Texas Instruments, TX, USA) to spatially modulate light to the appropriate sine wave patterns with three different phases (0, 2π/3, and 4π/3) and five spatial frequencies from 0 to 2.0 cm^−1^ at a resolution of 1280 × 800 pixels. Light from three high-power LEDs at 490 nm, 590 nm, and 656 nm was directed to the DMD through a liquid light guide (Mightex), with the LEDs and DMD being controlled remotely via MATLAB. The 490 nm LED excited Dox fluorescence and captured SFDI optical properties at its excitation peak, the 590 nm LED measured optical properties and Dox fluorescence at its emission peak, and the 656 nm light excited Porphyrin fluorescence.

The DMD module generated sinusoidal patterns and projected them through a 2.4 mm imaging fiber with 13,000 elements (Asahi Kasei, Tokyo, Japan) which passed through the fixed laparoscope to the distal end facing the imaged surface. A custom objective lens at the tip of the fiber served to collimate light from the fiber and homogeneously projected onto the tissue. Patterns from the DMD were reflected off the tissue and collected through the laparoscope optics, which includes the laparoscope (8912.43, R. Wolf, Vernon Hills, IL, USA), a zoom coupler (Accu-Beam, TTI Medical, CA, USA), two 30 mm achromatic lenses, a filter wheel, and an aperture (ThorLabs, NJ, USA) before reaching the EMCCD camera (1004 × 1002 pixels, Luca, Andor, Belfast, Ireland). The camera was focused over the entire area of the projected SFDI pattern at a 3.2 × 3.2 cm field of view (FOV). The optical design ensures an accurate sinusoidal projection for SFDI in a format using the components currently used in laparoscopic surgery.

For acquiring SFDI data, no optical filters were used, with the LED output centered on the respective 490 nm and 590 nm excitation and emission wavelengths of Doxorubicin. When imaging Doxorubicin fluorescence, a 530 nm long pass filter and a 593 ± 40 nm fluorescent filter were used to isolate Doxorubicin fluorescence from the excitation light. When imaging Porphyrin fluorescence, a 660 ± 10 nm bandpass filter was used to isolate the PoP excitation signal, and a 716 ± 40 nm fluorescence filter was used to separate PoP fluorescence. The fluorescent images were taken with a 6 s exposure time and a total of 12 s (including dark) at 16 × 16 camera binning, whereas the SFDI measurements were taken at 2 s exposure per projection, at 8 × 8 camera binning with a total time (3 phases × 5 frequency) of 30 s for each wavelength. For the SFDI measurements, cross-polarizers were built into the front of the laparoscope’s distal end to reduce spectral reflection. A structural frame acted as a mechanical stop to ensure that the object surface was no closer than the minimal effective working distance of the scope. Due to the divergence of light from the objective lenses, the frequency of the projected SFDI patterns will increase with distance, so the structural frame ensures that the target is always close to the optimal working distance. The optical absorption and scattering properties were extracted from the SFDI images by a modified frequency-domain Monte Carlo model from the measured reflectance data across multiple spatial frequencies as described previously but with a total of 15 images at 5 frequencies ranging from 0 to 2.0 cm^−1^.

### 4.3. System Calibration

The wide-field SFDI instrument was tested on ovarian tissue-mimicking phantoms with optical absorption (µ_a_) and scattering (µ_s_′) properties within the range of tissue at 660 nm, which is the common wavelength for PoP-based PDT. The calibration phantoms used in this experiment were fabricated based on absorption and scattering parameters from prior work [48]. Bulk optical parameters were quantified by fitting frequency-dependent reflectance data with modified frequency-domain diffusion models by using a reference phantom with known optical properties [47].

The laparoscopic SFDI system was characterized with multiple calibration phantoms made by titrating the optical properties ranging from µ_a_ = 0.5 cm^−1^ to 1.5 cm^−1^ and µ_s_′ = 10 cm^−1^ to 30 cm^−1^ at 490 nm and 590 nm. The mean percent error in quantifying the absorption parameter was always less than 10%. Fluorescence phantoms were prepared by titrating the Dox concentration, with a 0.5 mg/mL free-Dox solution used to acquire fluorescence values at 2, 4, 6, and 8 µg/mL in phantoms of varied optical properties. Reconstructed raw fluorescence values for concentration were used as a calibration curve to obtain absolute Dox concentrations as previously described [48].

### 4.4. Subcutaneous Tumor Culture in Mouse Model

Human epithelial ovarian adenocarcinoma cell lines SKOV3 were purchased from the American Type Culture Collection (ATCC, HTB-77, Manassas, VA, USA). The cells were maintained in McCoy’s 5A medium supplemented with 10% fetal bovine serum and 1% antibiotic-antimycotic. The cells were cultured at 37 °C in a humidified atmosphere of 95% air and 5% CO_2_ (*v*/*v*). The cells were routinely sub-cultured when 90% confluence was reached using 0.25% *w*/*v* trypsin-EDTA solution. The cell culture medium and supplements were purchased from Gibco-Life Technologies (Grand Island, NY, USA).

In vivo studies were conducted by the Institutional Animal Care and Use Committee (IACUC) of Wright State University and Stony Brook University-approved protocols. All animal care was performed following the relevant guidelines and regulations outlined in the “Guide for Care and Use of Laboratory Animals”.

Female athymic *nu*/*nu* (nude) mice were purchased from Charles River Laboratories. When they were 8–10 weeks old, they were injected subcutaneously (*sc*) with 10^7^ SKOV3 cells. The mice were then monitored for the growth of tumors, and tumor diameters (length, L and width, W) were measured with a caliper. Tumor volume was calculated using the following formula: (L × W^2^)/2. When the tumors reached approximately 9 mm in diameter, the mice were i.v. injected with 0.4 µg/mL Dox-PoP in 400 µL 5% dextrose solution. For imaging, the mice were placed on a heating pad and anesthetized with isoflurane. The injection was left to diffuse within the tumor for 1 h before fluorescence imaging with 660 nm LED through the 740 nm EMCCD filter. A treatment light at 660 nm was applied to the injection site for 5 min, after which fluorescence images were taken again using the same parameters.

### 4.5. Preparation of Long-Circulating Dox in PoP Liposomes

The details of our PoP Dox liposome formulation have been described in [38,49]. PoP-liposomes incorporated PoP photosensitizer in a recent study for the development of improved peptide-based cancer vaccines, underscoring the versatility of the drug delivery platform. Briefly, PoP was synthesized from pyro-lipid through the esterification of pyro with lyso-C16-PC using 1-ethyl-3-(3-dimethylaminopropyl) carbodiimide (EDC) and 4-dimethylaminopyridine (DMAP) in chloroform. The liposomes were formed by dissolving DSPC, DSPE-PEG2k, cholesterol, and PoP (molar ratio: 53:5:40:2) in ethanol (2 mL) at 60C, followed by the addition of 250 mM ammonium sulfate (8 mL, pH 5.5) at 60 °C. The 20 mg/mL lipid solution was extruded through a Lipex high-pressure lipid extruder with a 250 mM ammonium sulfate solution using polycarbonate membranes of 0.2, 0.1, and 0.08 µm pore size, sequentially stacked and passed through the extruder 10 times. Free ammonium sulfate was removed by overnight dialysis in a 10% sucrose solution with 10 mM histidine (pH 6.5) with 2 buffer exchanges over 24 h. Dox was loaded (2 mg/mL Dox: 0.35 mg/mL PoP) by incubating the liposomes at 60 °C for 1 h, achieving a loading efficacy of over 90% as confirmed by G-75 column elution assay. The self-assembly status and elution fraction of PoP-liposomes were tracked using 420 nm excitation and 670 nm emission, while Dox gel fractionation was detected using 480 nm excitation and 590 nm emission using a fluorescence plate reader (TECAN Safire, Männedorf, Switzerland).

**Figure 9:**
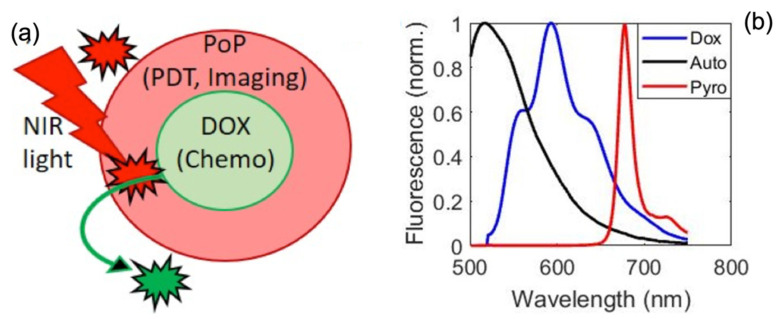
A liposomal “nano-balloon” consists of PoP and Dox components: (**a**) Schematic of Dox-PoP liposomes. NIR light activates PoP, triggering the release of Dox from the liposome. (**b**) The basis spectra of Doxorubicin (Dox), PoP (also called as Pyro), and autofluorescence (Auto) were used for quantifying fluorescence concentrations.

We used the fluorescence of the PoP in the Dox-PoP liposomes shown in red in Figure 9a as the primary imaging marker to estimate initial drug concentration. Before release, PoP fluorescence indicates the location of tumor areas, while post-release Dox fluorescence (triggered by structured NIR light, Figure 9b marks localized chemodrug (Dox) release [25]. Tissue optical properties enable fluorescence attenuation correction, and thereby the quantification of drug concentration in target tissues.

### 4.6. Dox Release Quantification and PoP Photobleaching by Laparoscopic SFDI in a Mouse Model

A recently sacrificed BALB/c mouse was acquired for simulated in vivo measurements. The mouse was placed on an imaging platform with the shaved injection site centered within the 3.2 × 3.2 cm FOV of the laparoscope. SFDI measurements at the 490 nm excitation and 590 nm emission wavelengths of Doxorubicin were performed before injection. The mouse was injected subcutaneously with 50 µL of a lightly scattering intralipid^®^ medium (µ_s_′ = 5 cm^−1^ at 490 nm) containing 23.1 µg/mL PoP liposomes, and SFDI measurements were performed again post-injection. A treatment light at 657 nm at a fluence rate of 330 mW/cm^2^ was directed onto a 1 cm area centered on the injection site, and fluorescence measurements were acquired every 1 min to assess drug release dynamics. PoP fluorescence images were acquired immediately before and after applying treatment light to assess photobleaching.

### 4.7. Attenuation-Compensated Fluorescence Analysis

By using SFDI in fluorescence imaging mode, photosensitizer (PS) fluorescence can allow the quantification of PS concentration by accurately compensating for light attenuation at both excitation and emission wavelengths. The quantification of PoP fluorescence concentration was performed using the Gardner model [50], which corrects the raw fluorescence signal by accounting for optical absorption (µ_a_) and scattering (µ_s_′) losses at both the excitation and emission wavelengths. In this model, the fluorescence correction factor, X_1D_(ex, em), is determined using the quantified optical parameters from SFDI measurements, where X_1D_(ex, em), represents the effective path length during the penetration of excitation light and the escape of emitted fluorescence from the tissue. The corrected fluorescence was then calculated as F_corr_ = F_raw_/X_1D_, where F_raw_ is the measured raw signal, X_1D_ is the correction factor, and F_corr_ is the fluorescence corrected for optical absorption, scattering, and light propagation. Using the calibration factor, the corrected fluorescence was translated into PoP concentrations. Fluorescence imaging can also be used for monitoring PDT response because PS fluorescence changes during PDT, and these changes may be indicative of PDT response.

To quantitatively compare tumor regions with surrounding normal tissue, an image analysis was performed using a hand-drawing tool function (*imfreehand*/*impoly*) in MATLAB (MathWorks, Inc., Natick, MA, USA). Regions of interest (ROIs) for both tumor and peripheral tissue were selected based on reflectance maps at 590, 625, 660, and 740 nm. Statistical metrics, including mean and standard deviation, for each ROI are summarized in a bar plot (Figure 3). The analysis revealed contrasts between the tumor and peripheral tissue, with the tumor ROIs exhibiting higher mean absorption parameters and lower mean scattering. Laparoscopic SFDI data was processed similarly, with ROIs pertinent to the injected drug area being selected for analysis at each fluorescence acquisition. The mean value of this area for each minute was used to determine Dox release kinetics, and images taken at the PoP fluorescence wavelength before and after light exposure were compared to assess PoP photobleaching. Data is summarized with Dox release leveling off in Figure 5d after sufficient light dosage, and PoP fluorescence decreasing due to photobleaching by Dox-PoP liposome light.

## 5. Conclusions

This study demonstrates the results of a wide-field laparoscopic SFDI system for the real-time intraoperative monitoring of Dox-PoP. The system accurately measures PoP photobleaching and Dox release kinetics by enabling quantitative fluorescence imaging and overcoming tissue absorption and scattering limitations. The observed PDT-mediated PoP photobleaching and light-triggered drug activation confirm the laparoscopic SFDI system’s capability to enhance targeted tumor treatment while minimizing off-target effects. Compared to traditional flexible endoscopic imaging, this approach offers larger FOV and precise optical property quantification, making it a promising tool for minimally invasive PDT and chemotherapeutic drug delivery. These findings highlight the potential of laparoscopic SFDI to improve CPT efficacy, ultimately advancing the real-time monitoring of cancer treatment. Future studies should investigate its application in clinical settings and explore its integration with other imaging modalities for comprehensive tumor characterization. The quantitative fluorescence imaging approach demonstrated here has strong potential for future clinical translation, enabling the real-time monitoring of drug release and photodynamic response in minimally invasive cancer surgeries. Beyond ovarian cancer, this technique may be adapted for image-guided chemophototherapy in other tumors such as brain, gastrointestinal, and lung, supporting broader clinical trials and precision treatment strategies.

## Data Availability

The data that support the findings of this study are available from the corresponding author upon reasonable request.

## References

[1] Azaïs H., Schmitt C., Tardivel M., Kerdraon O., Stallivieri A., Frochot C., Betrouni N., Collinet P., Mordon S. (2016). Assessment of the specificity of a new folate-targeted photosensitizer for peritoneal metastasis of epithelial ovarian cancer to enable intraperitoneal photodynamic therapy. A preclinical study. Photodiagnosis Photodyn. Ther..

[2] Longmire M., Kosaka N., Ogawa M., Choyke P.L., Kobayashi H. (2009). Multicolor in vivo targeted imaging to guide real-time surgery of HER2-positive micrometastases in a two-tumor coincident model of ovarian cancer. Cancer Sci..

[3] Sugiyama T., Nishida T., Komai K., Nishimura H., Yakushiji M. (1996). Comparison of CA 125 assays with abdominopelvic computed tomography and transvaginal ultrasound in monitoring of ovarian cancer. Int. J. Gynecol. Obstet..

[4] Chan J.K., Monk B.J., Cuccia D., Pham H., Kimel S., Gu M., Hammer-Wilson M.J., Liaw L.-H.L., Osann K., DiSaia P.J. (2002). Laparoscopic Photodynamic Diagnosis of Ovarian Cancer Using 5-Aminolevulinic Acid in a Rat Model. Gynecol. Oncol..

[5] Rizvi I., Celli J.P., Evans C.L., Abu-Yousif A.O., Muzikansky A., Pogue B.W., Finkelstein D., Hasan T. (2010). Synergistic enhancement of carboplatin efficacy with photodynamic therapy in a three-dimensional model for micrometastatic ovarian cancer. Cancer Res..

[6] van Dam G.M., Themelis G., Crane L.M., Harlaar N.J., Pleijhuis R.G., Kelder W., Sarantopoulos A., de Jong J.S., Arts H.J., van der Zee A.G. (2011). Intraoperative tumor-specific fluorescence imaging in ovarian cancer by folate receptor-α targeting: First in-human results. Nat. Med..

[7] Rose P.G., Faulhaber P., Miraldi F., Abdul-Karim F.W. (2001). Positive Emission Tomography for Evaluating a Complete Clinical Response in Patients with Ovarian or Peritoneal Carcinoma: Correlation with Second-Look Laparotomy. Gynecol. Oncol..

[8] Boussios S., Pentheroudakis G., Katsanos K., Pavlidis N. (2012). Systemic Treatment-Induced Gastrointestinal Toxicity: Incidence, Clinical Presentation and Management. Ann Gastroenterol..

[9] Mallidi S., Anbil S., Bulin A.-L., Obaid G., Ichikawa M., Hasan T. (2016). Beyond the Barriers of Light Penetration: Strategies, Perspectives and Possibilities for Photodynamic Therapy. Theranostics.

[10] Sunar U., Rohrbach D., Rigual N., Tracy E., Keymel K., Cooper M.T., Baumann H., Henderson B.H. (2010). Monitoring photobleaching and hemodynamic responses to HPPH-mediated photodynamic therapy of head and neck cancer: A case report. Opt. Express.

[11] Mo W., Rohrbach D.J., Sunar U. (2012). Imaging a photodynamic therapy photosensitizer in vivo with a time-gated fluorescence tomography system. J. Biomed. Opt..

[12] Ozturk M.S., Rohrbach D., Sunar U., Intes X. (2014). Mesoscopic Fluorescence Tomography of a Photosensitizer (HPPH) 3D Biodistribution in Skin Cancer. Acad. Radiol..

[13] Staropoli N., Ciliberto D., Botta C., Fiorillo L., Grimaldi A., Lama S., Caraglia M., Salvino A., Tassone P., Tagliaferri P. (2014). Pegylated liposomal doxorubicin in the management of ovarian cancer. Cancer Biol. Ther..

[14] Sunar U., Rohrbach D.J., Morgan J., Zeitouni N., Henderson B.W. (2013). Quantification of PpIX concentration in basal cell carcinoma and squamous cell carcinoma models using spatial frequency domain imaging. Biomed. Opt. Express.

[15] Major A.L., Rose G., Chapman C.F., Hiserodt J.C., Tromberg B.J., Krasieva T.B., Tadir Y., Haller U., Disaia P.J., Berns M.W. (1997). In VivoFluorescence Detection of Ovarian Cancer in the NuTu-19 Epithelial Ovarian Cancer Animal Model Using 5-Aminolevulinic Acid (ALA). Gynecol. Oncol..

[16] Celli J.P., Rizvi I., Blanden A.R., Massodi I., Glidden M.D., Pogue B.W., Hasan T. (2014). An imaging-based platform for high-content, quantitative evaluation of therapeutic response in 3D tumour models. Sci. Rep..

[17] Spring B.Q., Sears R.B., Zheng L.Z., Mai Z., Watanabe R., Sherwood M.E., Schoenfeld D.A., Pogue B.W., Pereira S.P., Villa E. (2016). A photoactivable multi-inhibitor nanoliposome for tumour control and simultaneous inhibition of treatment escape pathways. Nat. Nanotechnol..

[18] Wang S., Hüttmann G., Scholzen T., Zhang Z., Vogel A., Hasan T., Rahmanzadeh R. (2016). A light-controlled switch after dual targeting of proliferating tumor cells via the membrane receptor EGFR and the nuclear protein Ki-67. Sci. Rep..

[19] Zhong W., Celli J.P., Rizvi I., Mai Z., Spring B.Q., Yun S.H., Hasan T. (2009). In vivo high-resolution fluorescence microendoscopy for ovarian cancer detection and treatment monitoring. Br. J. Cancer.

[20] Azaïs H., Queniat G., Bonner C., Kerdraon O., Tardivel M., Jetpisbayeva G., Frochot C., Betrouni N., Collinet P., Mordon S. (2015). Fischer 344 Rat: A Preclinical Model for Epithelial Ovarian Cancer Folate-Targeted Therapy. Int. J. Gynecol. Cancer.

[21] Guyon L., Ascencio M., Collinet P., Mordon S. (2012). Photodiagnosis and photodynamic therapy of peritoneal metastasis of ovarian cancer. Photodiagnosis Photodyn. Ther..

[22] Allen T.M., Cullis P.R. (2013). Liposomal drug delivery systems: From concept to clinical applications. Adv. Drug Deliv. Rev..

[23] Li S.-D., Huang L. (2008). Pharmacokinetics and Biodistribution of Nanoparticles. Mol. Pharm..

[24] Kress J., Rohrbach D.J., Carter K.A., Luo D., Poon C., Aygun-Sunar S., Shao S., Lele S., Lovell J.F., Sunar U. (2017). A dual-channel endoscope for quantitative imaging, monitoring, and triggering of doxorubicin release from liposomes in living mice. Sci. Rep..

[25] Rohrbach D.J., Carter K.A., Luo D., Shao S., Aygun-Sunar S., Lovell J.F., Sunar U. (2024). Fluence rate-dependent kinetics of light-triggered liposomal doxorubicin assessed by quantitative fluorescence-based endoscopic probe. bioRxiv.

[26] Carter K.A., Shao S., Hoopes M.I., Luo D., Ahsan B., Grigoryants V.M., Song W., Huang H., Zhang G., Pandey R.K. (2014). Porphyrin–phospholipid liposomes permeabilized by near-infrared light. Nat. Commun..

[27] Luo D., Carter K.A., Razi A., Geng J., Shao S., Lin C., Ortega J., Lovell J.F. (2015). Porphyrin-phospholipid liposomes with tunable leakiness. J. Control. Release.

[28] Venugopal V., Park M., Ashitate Y., Neacsu F., Kettenring F., Frangioni J.V., Gangadharan S.P., Gioux S. (2013). Design and characterization of an optimized simultaneous color and near-infrared fluorescence rigid endoscopic imaging system. J. Biomed. Opt..

[29] Palmer G.M., Boruta R.J., Viglianti B.L., Lan L., Spasojevic I., Dewhirst M.W. (2010). Non-invasive monitoring of intra-tumor drug concentration and therapeutic response using optical spectroscopy. J. Control. Release.

[30] Bogaards A., Sterenborg H.J.C.M., Wilson B.C. (2007). In vivo quantification of fluorescent molecular markers in real-time: A review to evaluate the performance of five existing methods. Photodiagnosis Photodyn. Ther..

[31] Yang B., Sharma M., Tunnell J.W. (2013). Attenuation-corrected fluorescence extraction for image-guided surgery in spatial frequency domain. J. Biomed. Opt..

[32] Yang V.X.D., Muller P.J., Herman P., Wilson B.C. (2003). A multispectral fluorescence imaging system: Design and initial clinical tests in intra-operative Photofrin-photodynamic therapy of brain tumors. Lasers Surg. Med..

[33] Baran T.M., Foster T.H. (2013). Recovery of intrinsic fluorescence from single-point interstitial measurements for quantification of doxorubicin concentration. Lasers Surg. Med..

[34] Kim A., Khurana M., Moriyama Y., Wilson B.C. (2010). Quantification of in vivo fluorescence decoupled from the effects of tissue optical properties using fiber-optic spectroscopy measurements. J. Biomed. Opt..

[35] Zhadin N.N., Alfano R.R. (1998). Correction of the Internal Absorption Effect in Fluorescence Emission and Excitation Spectra from Absorbing and Highly Scattering Media: Theory and Experiment. J. Biomed. Opt. April..

[36] Sheng C., Pogue B.W., Wang E., Hutchins J.E., Hoopes P.J. (2004). Assessment of Photosensitizer Dosimetry and Tissue Damage Assay for Photodynamic Therapy in Advanced-stage Tumors. Photochem. Photobiol..

[37] Applegate M.B., Karrobi K., Angelo J.P., Austin W.M., Tabassum S.M., Aguénounon E., Tilbury K., Saager R.B., Gioux S., Roblyer D.M. (2020). OpenSFDI: An open-source guide for constructing a spatial frequency domain imaging system. J. Biomed. Opt..

[38] Luo D., Carter K.A., Razi A., Geng J., Shao S., Giraldo D., Sunar U., Ortega J., Lovell J.F. (2016). Doxorubicin encapsulated in stealth liposomes conferred with light-triggered drug release. Biomaterials.

[39] Bellnier D.A., Henderson B.W., Pandey R.K., Potter W.R., Dougherty T.J. (1993). Murine pharmacokinetics and antitumor efficacy of the photodynamic sensitizer 2-[1-hexyloxyethyl]-2-devinyl pyropheophorbide-a. J. Photochem. Photobiol. B.

[40] Georgakoudi I., Nichols M.G., Foster T.H. (1997). The Mechanism of Photofrin Photobleaching and Its Consequences for Photodynamic Dosimetry. Photochem. Photobiol..

[41] Sheng C., Jack Hoopes P., Hasan T., Pogue B.W. (2007). Photobleaching-based Dosimetry Predicts Deposited Dose in ALA-PpIX PDT of Rodent Esophagus. Photochem. Photobiol..

[42] Wilson B.C., Patterson M.S., Lilge L. (1997). Implicit and explicit dosimetry in photodynamic therapy: A New paradigm. Lasers Med. Sci..

[43] Zhou X., Pogue B.W., Chen B., Demidenko E., Joshi R., Hoopes J., Hasan T. (2006). Pretreatment photosensitizer dosimetry reduces variation in tumor response. Int. J. Radiat. Oncol. Biol. Phys..

[44] Angelo J.P., van de Giessen M., Gioux S. (2017). Real-time endoscopic optical properties imaging. Biomed. Opt. Express.

[45] van de Giessen M., Angelo J.P., Gioux S. (2015). Real-time, profile-corrected single snapshot imaging of optical properties. Biomed. Opt. Express.

[46] Angelo J., Vargas C.R., Lee B.T., Bigio I.J., Gioux S. (2016). Ultrafast optical property map generation using lookup tables. J. Biomed. Opt..

[47] Cuccia D.J., Bevilacqua F., Durkin A.J., Ayers F.R., Tromberg B.J. (2009). Quantitation and mapping of tissue optical properties using modulated imaging. J. Biomed. Opt..

[48] Kress J., Rohrbach D.J., Carter K.A., Luo D., Shao S., Lele S., Lovell J.F., Sunar U. (2015). Quantitative imaging of light-triggered doxorubicin release. Biomed. Opt. Express.

[49] Gordon A.N., Fleagle J.T., Guthrie D., Parkin D.E., Gore M.E., Lacave A.J. (2001). Recurrent Epithelial Ovarian Carcinoma: A Randomized Phase III Study of Pegylated Liposomal Doxorubicin Versus Topotecan. J. Clin. Oncol..

[50] Gardner C.M., Jacques S.L., Welch A.J. (1996). Fluorescence spectroscopy of tissue: Recovery of intrinsic fluorescence from measured fluorescence. Appl. Opt..

